# Structome-AlignViewer: On Confidence Assessment in Structure-Aware Alignments

**DOI:** 10.1093/gbe/evag004

**Published:** 2026-01-13

**Authors:** Ashar J Malik, Siying Mao, Philip Hugenholtz, David B Ascher

**Affiliations:** School of Chemistry and Molecular Biosciences, The University of Queensland, Brisbane, Australia; Australian Centre for Ecogenomics, The University of Queensland, Brisbane, Australia; Computational Biology and Clinical Informatics, Baker Heart and Diabetes Institute, Melbourne, Victoria, Australia; School of Chemistry and Molecular Biosciences, The University of Queensland, Brisbane, Australia; Australian Centre for Ecogenomics, The University of Queensland, Brisbane, Australia; School of Chemistry and Molecular Biosciences, The University of Queensland, Brisbane, Australia; Australian Centre for Ecogenomics, The University of Queensland, Brisbane, Australia; School of Chemistry and Molecular Biosciences, The University of Queensland, Brisbane, Australia; Australian Centre for Ecogenomics, The University of Queensland, Brisbane, Australia; Computational Biology and Clinical Informatics, Baker Heart and Diabetes Institute, Melbourne, Victoria, Australia

**Keywords:** structure-based alignment, protein structure comparison, alignment confidence scoring, deep structural homology, structural phylogenetics

## Abstract

Protein structure-based comparison provides a framework for uncovering deep evolutionary relationships that can escape conventional sequence-based approaches. Encoding three-dimensional protein structures using a simplified structure-aware alphabet can lead to compact, comparable strings that retain key spatial relationships. Although this enables comparison, structure-aware alignments can experience misaligned regions, particularly when comparing proteins with substantial divergence in fold architecture. To address this, a web-based resource, Structome-AlignViewer, is introduced in this work for evaluating the quality of structure-aware alignments through both spatial mapping of alignment columns to protein structures and quantitative confidence scoring. Confidence is computed from pairwise structural substitutions between adjacent inputs and normalized within each alignment to highlight relatively well-supported columns. To provide broader context, thousands of alignments from established structural classification systems were analyzed, allowing for an empirical comparative statistic to be derived to assess alignment quality. The option to exclude gap-rich regions enables users to refine alignments and focus on conserved structural cores. This approach provides an interpretable method for assessing structural alignment quality and supports more robust comparative and evolutionary analyses. Structome-AlignViewer is freely available at https://biosig.lab.uq.edu.au/structome_alignviewer/.

Significance Similarity between proteins is usually determined by aligning their amino-acid sequences, yet this sequence signal of homology fades as changes accumulate over evolutionary time-scales. Protein structures, however, retain evolutionary signal far longer. Foldseek encodes these shapes into structure-aware strings that support phylogenetic analysis through assembly of multiple-sequence structure-aware alignments. Researchers however still need to see how each column of the alignment corresponds to the position in the three-dimensional protein structure.

Structome-AlignViewer is a free web resource that fills this gap. It converts structures to their structure-aware sequence forms, builds the alignment, and then projects every column back onto the protein models using an interactive web-based protein structure visualizer, and assigning an easy-to-read confidence score to each position. Benchmarked across thousands of SCOP and CATH families, these scores highlight conserved cores and expose mis-aligned or gap-rich regions, letting biologists probe deep evolutionary relationships with far greater assurance.

## Introduction

Determining homology among biological macromolecules has long been central to unraveling the history of life on Earth. By identifying relationships among genes and proteins, homology inference provides the scaffolding upon which evolutionary biology, comparative genomics, and structural bioinformatics are built.

Traditionally, molecular sequence data have served as the primary source for detecting evolutionary relationships (Yang and Rannala 2012). While comparative sequence-based analysis has driven decades of discovery, it faces a fundamental limitation: as sequences diverge over deep evolutionary time, the homology signal progressively erodes, making it increasingly difficult to discern signal from noise. This loss of detectable similarity defines the so-called “twilight zone” (Rost 1999) of sequence comparison, beyond which many evolutionary relationships are missed.

Protein structure, in contrast, tends to be more robust to changes in the underlying amino acid sequence (Illergård et al. 2009; Lundin et al. 2012; Malik et al. 2020). It preserves key aspects of molecular function and folding architecture even as mutations accumulate and sequence identity diminishes. This robustness has made structural data an appealing alternative for recovering deeper evolutionary signals (Malik et al. 2020; Moi et al. 2025). A recent advance in defining a structural alphabet—the introduction of 3Di states used by Van Kempen et al. (2024)—has opened a new frontier. By converting protein structures into 3Di sequences, classical alignment and phylogenetic tools can be applied to this structure-aware alphabet, effectively extending the temporal reach of evolutionary inference (Puente-Lelievre et al. 2023; Malik et al. 2024).

This structure-informed evolutionary analysis generally follows a three-step process (Puente-Lelievre et al. 2023): (1) compile a dataset of proteins (Malik et al. 2023; Malik and Ascher 2025), (2) align their structure-aware sequences, and (3) build a phylogenetic tree from the resulting alignment (Puente-Lelievre et al. 2023). As promising as this approach is, the novelty of the method raises important open questions about how best to apply and interpret the inference.

One key aspect, of the many, that warrants a deeper exploration is the quality of the alignment itself where even slight column misplacements can distort phylogenetic inference by skewing comparative estimates and resulting tree topologies (Hall 2005; Cantarel et al. 2006; Loytynoja and Goldman 2008). While structure-aware strategies incorporate three-dimensional context, users still require straightforward ways to assess per-column reliability and to map these regions back to structures for manual review. Tools such as FoldMason Gilchrist et al. (2024) address this challenge using the MSA-LDDT score, a metric that measures the conservation of local inter-atomic distances across aligned residues. However, a remaining gap is the ability to seamlessly link alignment columns to an interactive 3D structural view for intuitive inspection. Without such integration, refining this new class of alignments remains difficult, and downstream maximum-likelihood or other statistical phylogenetic methods risk receiving systematically biased inputs.

In this work, Structome-AlignViewer, an interactive, web-based platform is introduced that addresses this challenge by providing a fast and user-friendly environment to compute, inspect, and assess the quality of structure-aware alignments. The platform not only links alignment columns directly to 3D structures through an interactive and user-friendly interface, but also incorporates a statistical framework to quantify both per-column and overall alignment confidence. To contextualize these confidence values, an empirical alignment analysis across structurally homologous groups defined in SCOP Fox et al. (2014); Chandonia et al. (2022) and CATH Sillitoe et al. (2021) was carried out, yielding a background distribution of confidence scores. This distribution serves as a reference for assessing the significance of average confidence values in user-generated alignments. Similar to MSA-LDDT, our confidence score functions as a practical proxy for alignment reliability. Because it is computed from the same substitution matrix used by the aligner, it is conceptually related to the classical sum-of-pairs framework familiar from multiple sequence alignment. Structome-AlignViewer is alignment-method agnostic: it supports ClustalW-3Di, MAFFT-3Di, and also accepts user-provided MSAs, ensuring flexibility while making the interpretation of confidence scores explicit.

Overall, this resource is intended to provide more transparency for users carrying out structure-based phylogenetic analysis, and hence is intended to be an important advance in this area.

## Results

### Background Confidence Distributions from SCOP and CATH

**Fig. 1.**
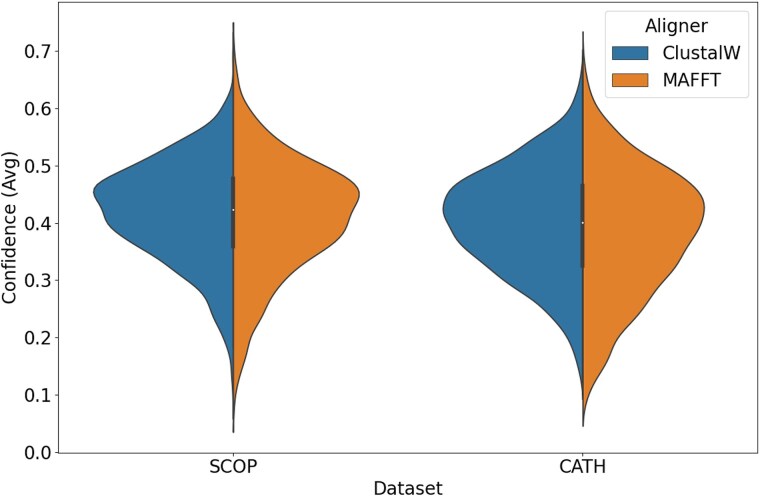
Distribution of average confidence scores for SCOP and CATH alignments, comparing results generated by ClustalW and MAFFT. Scores are calculated over the full, untrimmed alignments. The split violin plot shows that the confidence distributions are broadly similar for both alignment methods across the two datasets.

To contextualize the confidence scores computed for user-submitted alignments, background distributions were determined from the curated SCOP and CATH datasets using both the ClustalW and MAFFT alignment algorithms. These distributions serve as the statistical reference against which Z-scores are calculated. Figure 1 shows the resulting distributions of the average confidence scores, calculated over the full, untrimmed alignments. The broad similarity between the distributions is an expected outcome, as the confidence score calculation is analogous to the sum-of-pairs metric used internally by the alignment algorithms, and both methods were guided by the same 3Di substitution matrix. This result supports the inclusion of both algorithms in this resource, offering users a choice between a classic progressive alignment and a more modern iterative refinement approach, with the assurance that both produce alignments of comparable quality under this scoring framework. Additionally as expected, a strong negative correlation was observed between the overall gap fraction of an alignment and its average confidence score for both methods (see Supplementary Materials), reinforcing that trimming gap-rich columns is an effective strategy for refining an alignment to its conserved structural core. The trimmed alignments can therefore be interpreted as a refined subset, potentially more informative and suitable for downstream applications.

### Utility of the Confidence Score: A Case Study

To provide quantitative and actionable guidance on the utility of the confidence score, a case study was performed using a small, challenging dataset of divergent globins with a known ground truth. The set comprised pairs of proteins from all three domains of life, for which the expected ground truth is the recovery of three monophyletic pairs corresponding to each domain. Two inverse experiments were carried out to test the hypothesis that the true phylogenetic signal is concentrated in the high-confidence regions of the 3Di alignment.

**Fig. 2.**
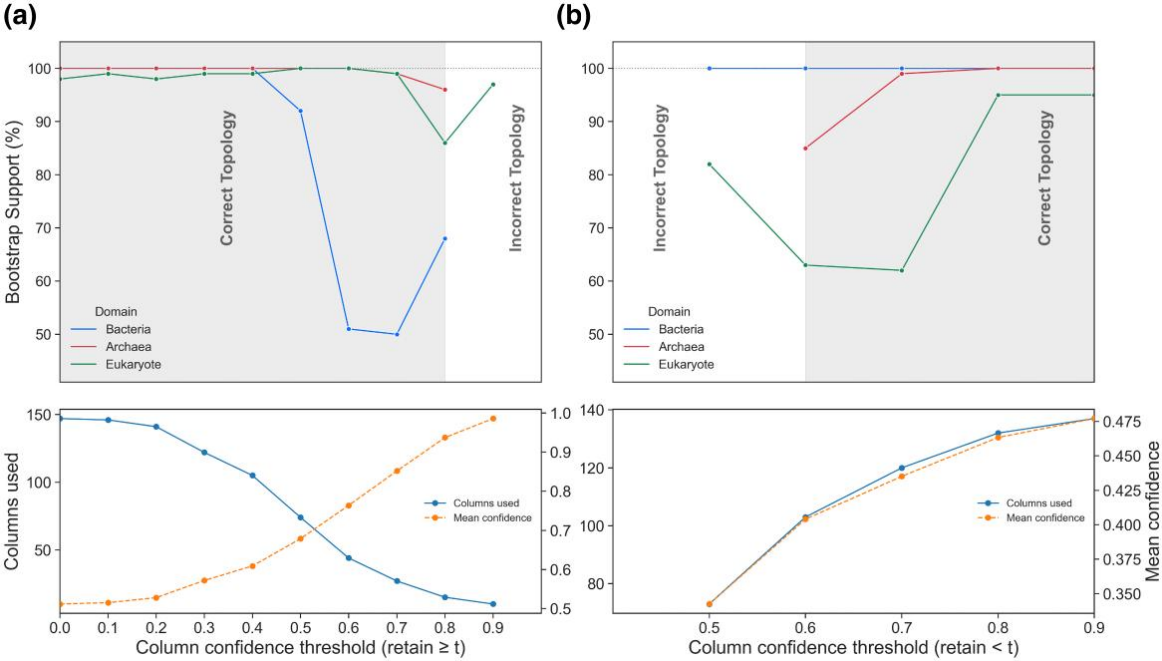
Phylogenetic signal stability and alignment composition across confidence-based trimming thresholds for the globin case study. **Top left** (robustness / inclusion sweep): Bootstrap support for the three ground-truth clades (Bacteria, Archaea, Eukaryote) vs. column confidence threshold *t* (retain ≥t). The grey band marks thresholds that recover the correct overall topology. **Top right** (negative control / exclusion sweep): the same, but columns retained have confidence <t; removing high-confidence sites rapidly collapses support and disrupts the correct topology. **Bottom left**: for the inclusion sweep, alignment size (columns used; left axis) and mean confidence of the retained columns (right axis) across *t*. **Bottom right**: the same metrics for the exclusion sweep. Panels within each column share the same *x*-axis. X-axes indicate the column confidence threshold as “retain ≥t” (inclusion) or “retain <t” (exclusion).

First, in a “robustness test”, the lowest-confidence columns were progressively removed from the alignment. As shown in Fig. 2a, the correct topology, with all three pairs resolved as monophyletic, was consistently recovered with high bootstrap support (>95%). This demonstrated that the phylogenetic signal was robust to the removal of alignment noise, holding true even when the alignment was trimmed to as few as 15 columns.

Second, in a “negative control” experiment, inverse exploration was carried out where the highest-confidence columns were progressively removed, leaving only the regions of lowest confidence for tree inference. As shown in Fig. 2b, this had a considerable effect. While alignments containing columns with confidence scores up to 0.6 still recovered the correct topology, the signal began to degrade rapidly as more high-confidence columns were removed. By the point where only columns with a confidence score below 0.5 remained, the tree topology was lost entirely, with the archaeal pair incorrectly resolved as non-monophyletic.

This dual experiment provides strong, actionable evidence that the evolutionary signal is indeed concentrated in the high-confidence regions. It demonstrates that the confidence score can be used not only to refine alignments by removing noise but also to rigorously diagnose the stability of a phylogenetic hypothesis. The full results of the trimming sweep, including bootstrap values at each confidence cutoff, are provided in the Supplementary Materials.

## Discussion

### Assessing Alignments using the Confidence Metric

Any structural alignment carried out for the purpose of determining homology, aims to identify equivalent positions across proteins compared. These equivalent positions then form the backbone of evolutionary comparison, delineating how folds are conserved, diverge, or adapt across the course of their respective evolutionary trajectories. Simply encoding 3D structures into structure-aware sequences before comparison does not necessarily guarantee a better alignment. As proteins diverge, segments may be introduced, which although part of a common fold may lack clear structural correspondence across the proteins, becoming especially pronounced when aligning distantly related proteins.

To determine if the alignment is reliable, the resource developed as part of this work, Structome-AlignViewer, offers two complementary layers of inspection. First, it enables users to directly map alignment columns back onto 3D structures which are rendered using the Mol${\;}^{*}$ protein structure viewer. This spatial mapping helps assess whether incompatible structure-aware characters have been aligned. Secondly, the confidence score brings statistical context and provides a way to evaluate alignment quality against the background alignments generated from curated SCOP and CATH datasets.

The confidence metric derived per alignment originates from the same substitution matrix used during the alignment process. This creates a conceptual dependence between the alignment and its confidence assessment, and in particular makes the metric related to the classic sum-of-pairs used to evaluate the quality of a multiple sequence alignment by the aligners during the alignment generation process. While not identical in formulation, both rely on scoring residue pairs within columns using the same substitution matrix. Accordingly, the confidence score introduced in this work is best understood as a measure of internal consistency: the alignment aims to optimally overlap structure-aware sequences, while the confidence score provides an additional per-column view of where the aligner itself finds strong or weak support. Although the scoring and alignment frameworks differ mathematically, they remain unified in vocabulary and logic, making the confidence score a natural extension of the alignment rather than an independent benchmark.

Additionally, confidence values are scaled to highlight the best and worst aligned columns, reflecting relative structural consistency rather than an absolute quality metric. Within each column, both identical and mismatched structure-aware characters are scored according to the structural alphabet’s substitution matrix. Normalization of all raw scores to a [0,1] range based on the alignment’s highest- and lowest-scoring columns not only captures whether residues agree but also how strongly they agree. As a result, columns that outperform the alignment’s average become visibly more trustworthy, whereas weakly supported regions are readily flagged.

To add more context, the average normalized confidence score is computed and compared to the distributions of the same metric derived from thousands of SCOP and CATH alignments. Although each alignment is normalized independently, these averages form meaningful empirical distributions. The resulting Z-scores allow users to contextualize their alignment within the known homologous families. While this score does not predict phylogenetic correctness, it offers a principled and statistically grounded way to assess alignment quality, and can help guide decisions about trimming, weighting, or downstream analysis.

### Limitations of the Confidence Metric

It must be emphasized that alignment confidence is not equivalent to phylogenetic confidence. While a reliable alignment is essential for downstream tree construction, high average alignment confidence does not imply correctness of the inferred topology. Many other factors—substitution model choice, rate heterogeneity, long-branch attraction, and sampling—can influence phylogenetic inference independently of alignment quality.

The purpose of Structome-AlignViewer is to provide users with a clearer view of how well their alignment represents conserved structural signals. Properly interpreted, confidence scores can guide alignment refinement without overextending their meaning whereas over-interpreting the score beyond its intended scope risks conflating alignment quality with evolutionary truth. This distinction should remain critical for all analyses involving this metric.

### Utility of the Confidence Score

While the globin case study provided a clear ‘ground truth’ against which the trimming strategy could be validated, such a reference is often unavailable in exploratory research. For this reason, the search for a single, universal confidence cutoff is not advocated.

Instead, the confidence score is presented as a tool for exploratory analysis. A sensitivity analysis can be conducted by performing a trimming sweep, similar to the one demonstrated in the case study. This process allows the stability of key nodes in a phylogeny to be assessed, differentiating between relationships that are robustly supported by the core structural signal and those that are sensitive to the inclusion of more variable, lower-confidence regions. In this context, the confidence score is transformed from a simple filter into a diagnostic tool for understanding how alignment ambiguity impacts phylogenetic inference for a specific set of proteins.

## Conclusion

Structome-AlignViewer, the resource introduced in this work, provides a robust framework for users to confidently assess structure-aware sequence alignments. It achieves this using two complementary methods. It allows visual inspection of alignment columns and additionally associates a confidence score with these columns. This combination allows users to assess the overall reliability of an alignment and identify regions that are well aligned across the protein structures compared. Additionally, to ensure consistency, the confidence score is calculated using the same substitution matrix employed for generating the alignment and is further normalized to facilitate comparisons both within a single alignment, and for computing averages which are then scored against extensive SCOP and CATH-derived distributions to provide valuable context.

While the confidence score, developed in this work, is not intended to be used toward assessing phylogenetic accuracy, it does open valuable avenues for further refinement. One immediate application is alignment pruning, i.e. removing poorly supported columns prior to tree inference. For example, in the case of non-parametric bootstrapping eliminating noisy positions could enhance signal-to-noise ratio and lead to reduced uncertainty in inferred trees such as demonstrated in the included case study.

As structure-based phylogenetics matures, Structome-AlignViewer will be instrumental in enhancing the reliability of structural comparisons carried out using structure-aware sequences and the evolutionary conclusions drawn from them.

## Methods

### Structure-aware Sequence Generation

This work uses Foldseek to generate structure-aware sequences of proteins using their respective 3D structures. Foldseek assigns each amino acid in the protein structure a structure-aware character based on the tertiary interactions made by the respective residue. This results in a sequence of structure-aware characters, which collectively are referred to as a structure-aware sequence in this work.

### Aligning Structure-aware Sequences

The alignment of structure-aware sequences was carried out using ClustalW2 (Larkin et al. 2007) and MAFFT (Katoh and Standley 2013), applying conventional multiple sequence alignment algorithms to the structure-aware representation. A custom substitution matrix (Van Kempen et al. 2024), included with Foldseek, was provided as input to both alignment engines, necessary as a distinction must be made between amino acids and structure-aware characters.

### Per-Column Confidence Metric

To assess alignment quality, a per-column confidence score $C_{j}$ is computed for each alignment column *j* based on the sum of substitution scores between adjacent sequences in the alignment (see supplementary data for examples). A complete all-versus-all sum would scale quadratically with sequence count; the adjacent-pair scheme is reasoned to retain linear complexity and, because the alignment order is fixed within this resource, it would yield deterministic confidence scores for a given protein set. Therefore, for each column, all adjacent sequence pairs are considered:


$$C_{j}=\sum_{i=1}^{N-1}S(r_{i},r_{i+1})$$

where:

- *N* is the number of sequences in the alignment,
-
$r_{i}$ and $r_{i+1}$ are the structure-aware characters from adjacent sequences at column *j*,
- S($r_{i}$,$r_{i+1}$) is the substitution score for that pair, obtained from the Foldseek substitution matrix.

Adjacent pairs involving gaps are excluded from analysis. The resulting raw column scores capture local structural coherence among neighboring sequences.

To normalize these values within each alignment, min-max scaling is applied across all columns:


$${\hat{C}}_{j}=\frac{C_{j}-C_{\min}}{C_{\max}-C_{\min}}$$

where $C_{\min}$ and $C_{\max}$ are the minimum and maximum raw confidence scores across all columns in the alignment. The resulting normalized score ${\hat{C}}_{j}$ lies in the range [0,1] and reflects the strength of structural agreement for each column relative to the weakest and strongest-aligned positions in the same alignment.

An average of all normalized column scores is also computed per alignment:


$${\hat{C}}_{\mathrm{avg}}=\frac{1}{L}\sum_{\;j=1}^{L}{\hat{C}}_{j}$$

where *L* is the total number of columns. This serves as a representative measure of overall alignment confidence.

### Interpretation of the Confidence Metric

To interpret confidence values statistically, benchmark datasets were prepared based on SCOP and CATH family groupings, adapted from previous work (Malik et al. 2024). To manage computational demands while prioritizing the most complete structural models, any family containing more than 300 members was subsampled by selecting its 300 longest sequences; families with 300 or fewer members were retained in their entirety. Structure-aware sequences for this curated set of families were then aligned using both the ClustalW2 and MAFFT alignment engines. For the final benchmark analysis, two thresholds were applied to these alignments:

- alignments comprising at least 10 structures, and
- alignments with a final length of at least 100 columns.

This resulted in final benchmark sets of 2,116 (ClustalW) and 2,111 (MAFFT) alignments for SCOP, and 1,964 (ClustalW) and 1,960 (MAFFT) alignments for CATH. Confidence scores were calculated for each alignment, and the corresponding means and standard deviations were determined for both classification systems for both alignment programs.

Z-scores reported on the results page on Structome-AlignViewer are derived from these distributions and computed separately for SCOP and CATH, for each of the alignment methods. The average of the two Z-scores ($Z_{\mathrm{avg}}$) is used to assign a qualitative confidence category for each alignment (see Table 1). This approach offers users a way to interpret alignment quality in a statistically meaningful way.

**Table 1.** Interpretation of Z-score-based alignment confidence levels, based on statistical rarity.

Z-score Range	Interpretation
Z≥2	Statistically Rare Alignment
1≤Z<2	Above Average, Less Common
−1<Z<1	Common, Expected Range
−2<Z≤−1	Below Average, Less Common
Z≤−2	Statistically Rare Alignment

### Implementation of the Web Interface

The Structome-AlignViewer resource is implemented using Flask, Docker and Nginx. The application provides a flexible input system with four distinct modes: (1) submission of a list of RCSB PDB accessions and chain identifiers; (2) submission of protein sequences in FASTA format, for which 3D structures are predicted using the ESMFold API; (3) upload of a Zip archive containing custom, user-provided single chain structure files in mmCIF format; and (4) direct input of a pre-computed multiple structure alignment in Clustal format. For workflows requiring de novo alignment, users can select between the ClustalW2 and MAFFT algorithms. Given compute limitations, a user is limited to 50 input structures per analysis.

For jobs involving 3D structures, the primary result page uses the Mol${\;}^{*}$ (Sehnal et al. 2021) viewer to display protein structures. The RCSB 1D feature viewer (Segura et al. 2020) shows the alignment, and the two components are interactively linked, allowing users to click an alignment column and instantly view the corresponding residue highlighted in the 3D viewer. A drop-down menu allows users to toggle between the different structures. Confidence scores are also encoded as B-factors in the rendered structures, enabling fast, intuitive color-based visualization of reliability (blue for low confidence, red for high). The page also presents a detailed “Job Information” panel, which includes metadata about the input type and alignment method, and a bar chart of column statistics (total, valid and dropped columns; where dropped columns are those with >50% gap content). For the pre-existing alignment input method, a streamlined results page is presented that omits the 3D structure viewer to focus solely on the alignment and its statistics.

Structome-AlignViewer also implements a Gblocks-style trimming option (Talavera and Castresana 2007), which removes alignment columns with >50% gap content. Raw structure-aware sequences for all structures, along with both full and trimmed alignments, and a guide tree/dendrogram are available for download in a single compressed archive.

Biopython is used both on Structome-AlignViewer and for other analysis included in this work to parse and analyze alignments and load confidence values as B-factors in the respective protein structures for subsequent visualization in the Mol${\;}^{*}$ viewer.

### Case Study: Phylogenetic Analysis of Divergent Globins

To quantitatively assess the utility of the confidence score, a case study was performed on a small dataset of six divergent globin domains with a well-established phylogeny, providing a clear “ground truth” against which to compare results. This dataset, a subset of one currently compiled for Structome-DeepRoots (Malik et al., unpublished), comprised representative pairs of sequences from all three domains of life (Eukaryote: *T. newnesi*, PDB 1T1N; Archaea: *A. pernix*, PDB 7UTE; Bacteria: *C. aurantiacus*, PDB 5D1V). This dataset was specifically chosen to provide a clear ground truth topology: it includes two evolutionarily diverged chains (alpha and beta hemoglobin) from the eukaryote, whose close relationship should be recovered, alongside two homodimeric proteins from the archaeal and bacterial representatives, whose identical chains are expected to form perfect monophyletic pairs.

The PDB and chain identifiers were submitted to Structome-AlignViewer using the “By PDB ID” input mode, and an initial alignment was generated using the ClustalW method. The corresponding per-column confidence scores were also obtained from the application’s output. Custom in-house Python scripts were then used to perform two controlled trimming experiments. First, in a “robustness test”, a series of alignments were generated by progressively removing the lowest-confidence columns. Second, in a “negative control”, alignments were generated by progressively removing the highest-confidence columns.

Maximum likelihood phylogenetic trees were inferred from each of these alignments using IQ-TREE with ultra-fast bootstrap. The resulting tree topologies and bootstrap support values were then programmatically analyzed using the DendroPy library to quantify the effect of the trimming on phylogenetic accuracy. The final results were plotted using the Matplotlib and Seaborn libraries.

## Supplementary Material

evag004_Supplementary_Data

## Data Availability

Structome-AlignViewer is freely available as a web application at: https://biosig.lab.uq.edu.au/structome_alignviewer/ For users wishing to perform large-scale batch analyses or process proteins locally, a standalone command-line interface (CLI) implementing the core methods described in this paper is publicly available at the following Bitbucket repository: https://bitbucket.org/proteinmechanic/structome_alignviewer/.
