# Supplementary material for: Structome-AlignViewer: On Confidence Assessment in Structure-Aware Alignments
Source: Genome Biol Evol. 2026 Jan 13;18(1):evag004. doi: 10.1093/gbe/evag004 (PMC12835816; doi:10.1093/gbe/evag004)
Supplement: evag004_Supplementary_Data [file evag004_supplementary_data.pdf]

# Structome-AlignViewer: On Confidence Assessment in Structure-Aware Alignments

Ashar J. Malik[1,2,3,*], Siying Mao[1,2], Philip Hugenholtz[1,2], and David B. Ascher[1,2,3,*]

[1]School of Chemistry and Molecular Biosciences, The University of Queensland, Brisbane, Australia

[2]Australian Centre for Ecogenomics, The University of Queensland, Brisbane, Australia

[3]Computational Biology and Clinical Informatics, Baker Heart and Diabetes Institute, Melbourne, Victoria, Australia

[*]Correspondence to Ashar J. Malik: ashar.malik@uq.edu.au, David B. Ascher: d.ascher@uq.edu.au

# Supplementary Example: Understanding confidence score using a toy example

To illustrate how per-column confidence scores are normalised within an alignment, a toy example involving 10 columns from a hypothetical structure-aware sequence alignment is used. Each column is scored based on the sum of substitution scores between adjacent sequence pairs, using the substitution matrix.

## Raw Confidence Scores

The raw confidence score $C_j$ for each column is computed by summing the substitution scores between adjacent sequence pairs in that column. Let us assume the following raw scores:

| **Column** | **Raw Score** ($C_j$) |
|---|---|
| 1 | $-5.0$ |
| 2 | $-1.5$ |
| 3 | $-1.0$ |
| 4 | $-2.0$ |
| 5 | $-1.3$ |
| 6 | $-0.8$ |
| 7 | $-1.2$ |
| 8 | $-1.1$ |
| 9 | $-0.5$ |
| 10 | 0.0 |

Table S1: Raw confidence scores per column based on adjacent sequence pair substitution sums.

## Min-Max Normalisation

Min-max normalisation is applied to rescale all scores between 0 and 1:

$$\hat{C}_j = \frac{C_j - C_{\min}}{C_{\max} - C_{\min}}$$

with:

$$C_{\text{min}} = -5.0, \quad C_{\text{max}} = 0.0$$

| **Column** | **Normalised Score ($\hat{C}_j$)** |
|---|---|
| 1 | 0.00 |
| 2 | 0.70 |
| 3 | 0.80 |
| 4 | 0.60 |
| 5 | 0.74 |
| 6 | 0.84 |
| 7 | 0.76 |
| 8 | 0.78 |
| 9 | 0.90 |
| 10 | 1.00 |

Table S2: Normalised confidence scores per column.

# Interpretation

This example highlights how min-max normalisation allows confidence values to be interpreted in relative terms within the alignment. Although columns 2–10 vary in their raw scores, they all score considerably higher than column 1, which contains structurally incoherent residue pairs. Consequently, even moderately supported columns (e.g., column 4) receive high normalised scores (0.6), because they are much more coherent than the worst column.

- The lowest scoring column ($C_1$) is mapped to 0.
- The highest scoring column ($C_{10}$) is mapped to 1.
- All other columns are scaled between these two extremes.

This approach ensures that confidence scores are contextualized within the alignment, and motivates the use of empirical benchmarking (e.g., via SCOP and CATH) to interpret how strong a given alignment is compared to structurally curated families.

# Supplementary Example: Understanding Confidence Scores from a Real Alignment

To illustrate how the confidence scoring system works in practice, a real example alignment of five structures is used from which five aligned columns are drawn. Confidence scores are computed using pairwise substitution values drawn from the Foldseek substitution matrix. This matrix defines substitution preferences between 20 structural states and is used both during alignment and confidence evaluation.

The substitution matrix is shown below:

| | A | C | D | E | F | G | H | I | K | L | M | N | P | Q | R | S | T | V | W | Y | X |
|---|---|---|---|---|---|---|---|---|---|---|---|---|---|---|---|---|---|---|---|---|---|
| A | 6 | -3 | 1 | 2 | 3 | -2 | -2 | -7 | -3 | -3 | -10 | -5 | -1 | 1 | -4 | -7 | -5 | -6 | 0 | -2 | 0 |
| C | -3 | 6 | -2 | -8 | -5 | -4 | -4 | -12 | -13 | 1 | -14 | 0 | 0 | 1 | -1 | 0 | -8 | 1 | -7 | -9 | 0 |
| D | 1 | -2 | 4 | -3 | 0 | 1 | 1 | -3 | -5 | -4 | -5 | -2 | 1 | -1 | -1 | -4 | -2 | -3 | -2 | -2 | 0 |
| E | 2 | -8 | -3 | 9 | -2 | -7 | -4 | -12 | -10 | -7 | -17 | -8 | -6 | -3 | -8 | -10 | -10 | -13 | -6 | -3 | 0 |
| F | 3 | -5 | 0 | -2 | 7 | -3 | -3 | -5 | 1 | -3 | -9 | -5 | -2 | 2 | -5 | -8 | -3 | -7 | 4 | -4 | 0 |
| G | -2 | -4 | 1 | -7 | -3 | 6 | 3 | 0 | -7 | -7 | -1 | -2 | -2 | -4 | 3 | -3 | 4 | -6 | -4 | -2 | 0 |
| H | -2 | -4 | 1 | -4 | -3 | 3 | 6 | -4 | -7 | -6 | -6 | 0 | -1 | -3 | 1 | -3 | -1 | -5 | -5 | 3 | 0 |
| I | -7 | -12 | -3 | -12 | -5 | 0 | -4 | 8 | -5 | -11 | 7 | -7 | -6 | -6 | -3 | -9 | 6 | -12 | -5 | -8 | 0 |
| K | -3 | -13 | -5 | -10 | 1 | -7 | -7 | -5 | 9 | -11 | -8 | -12 | -6 | -5 | -9 | -14 | -5 | -15 | 5 | -8 | 0 |
| L | -3 | 1 | -4 | -7 | -3 | -7 | -6 | -11 | -11 | 6 | -16 | -3 | -2 | 2 | -4 | -4 | -9 | 0 | -8 | -9 | 0 |
| M | -10 | -14 | -5 | -17 | -9 | -1 | -6 | 7 | -8 | -16 | 10 | -9 | -9 | -10 | -5 | -10 | 3 | -16 | -6 | -9 | 0 |
| N | -5 | 0 | -2 | -8 | -5 | -2 | 0 | -7 | -12 | -3 | -9 | 7 | 0 | -2 | 2 | 3 | -4 | 0 | -8 | -5 | 0 |
| P | -1 | 0 | 1 | -6 | -2 | -2 | -1 | -6 | -6 | -2 | -9 | 0 | 4 | 0 | 0 | -2 | -4 | 0 | -4 | -5 | 0 |
| Q | 1 | 1 | -1 | -3 | 2 | -4 | -3 | -6 | -5 | 2 | -10 | -2 | 0 | 5 | -2 | -4 | -5 | -1 | -2 | -5 | 0 |
| R | -4 | -1 | -1 | -8 | -5 | 3 | 1 | -3 | -9 | -4 | -5 | 2 | 0 | -2 | 6 | 2 | 0 | -1 | -6 | -3 | 0 |
| S | -7 | 0 | -4 | -10 | -8 | -3 | -3 | -9 | -14 | -4 | -10 | 3 | -2 | -4 | 2 | 6 | -6 | 0 | -11 | -9 | 0 |
| T | -5 | -8 | -2 | -10 | -3 | 4 | -1 | 6 | -5 | -9 | 3 | -4 | -4 | -5 | 0 | -6 | 8 | -9 | -5 | -5 | 0 |
| V | -6 | 1 | -3 | -13 | -7 | -6 | -5 | -12 | -15 | 0 | -16 | 0 | 0 | -1 | -1 | 0 | -9 | 3 | -10 | -11 | 0 |
| W | 0 | -7 | -2 | -6 | 4 | -4 | -5 | -5 | 5 | -8 | -6 | -8 | -4 | -2 | -6 | -11 | -5 | -10 | 8 | -6 | 0 |
| Y | -2 | -9 | -2 | -3 | -4 | -2 | 3 | -8 | -8 | -9 | -9 | -5 | -5 | -5 | -3 | -9 | -5 | -11 | -6 | 9 | 0 |
| X | 0 | 0 | 0 | 0 | 0 | 0 | 0 | 0 | 0 | 0 | 0 | 0 | 0 | 0 | 0 | 0 | 0 | 0 | 0 | 0 | 0 |

The following alignment represents five consecutive columns from five aligned protein structures. Each entry corresponds to a structure-aware character assigned to a residue by Foldseek. The alignment is shown in matrix format, where each row is a sequence and each column corresponds to a structurally aligned position. This block is centered within a longer alignment and represents columns $i$ through $i+4$:

| **Sequence** | **Col** $i$ | **Col** $i$+1 | **Col** $i$+2 | **Col** $i$+3 | **Col** $i$+4 |
|---|---|---|---|---|---|
| S1 | P | V | L | A | K |
| S2 | P | V | L | A | K |
| S3 | V | P | L | Q | K |
| S4 | V | P | L | Q | K |
| S5 | D | V | Q | G | W |

Table S3: Extract from a structure-based alignment showing five columns of a longer alignment.

For each alignment column, confidence scores are computed based on pairwise substitution values between adjacent sequence rows (e.g., S1–S2, S2–S3, etc.). These scores are summed to produce a raw confidence value $C_j$ for each column. Min-max normalisation is then applied across all five columns to produce normalised confidence values $\hat{C}_j$ in the range $[0, 1]$. The average of these normalised values is reported as $\hat{C}_{\text{avg}}$, representing the overall alignment confidence.

| **Column** | **Residue Pairs** | **Pair Scores** | $\mathbf{C_j}$ | $\hat{\mathbf{C}}_\mathbf{j}$ |
|---|---|---|---|---|
| Col $i$ | (P,P), (P,V), (V,V), (V,D) | 4, 0, 3, -3 | 4 | 0.00 |
| Col $i$+1 | (V,V), (V,P), (P,P), (P,V) | 3, 0, 4, 0 | 7 | 0.11 |
| Col $i$+2 | (L,L), (L,L), (L,L), (L,Q) | 6, 6, 6, 2 | 20 | 0.57 |
| Col $i$+3 | (A,A), (A,Q), (Q,Q), (Q,G) | 6, 1, 5, -4 | 8 | 0.14 |
| Col $i$+4 | (K,K), (K,K), (K,K), (K,W) | 9, 9, 9, 5 | 32 | 1.00 |

Table S4: Raw and normalised confidence scores for a real alignment example across five columns. $\hat{C}_j$ is computed by min-max normalisation with $C_{\text{min}} = 4$ and $C_{\text{max}} = 32$. The average normalised confidence across columns is $\hat{C}_{\text{avg}} = 0.364$.

This example highlights how confidence scores reflect local structural agreement among adjacent sequences and are scaled based on alignment-internal variation. The normalised values do not reflect absolute correctness but instead provide a relative measure to highlight which columns are most structurally consistent within the alignment.

# Supplementary Example: Alignment Statistics

The following section shows the complete alignment for SCOP family (accession d.12.1.3), based on structure-aware sequences.

```
2v94_A_1-93       DDKAWDDWDQDPVLAKIKTKIKDQDPPDDADALQVVLVCVCVVVVADSLFKFWDDWADDP
2v94_B_1-93       DDKAWDDWDQDPVLAKIKTKIKDADPPDDDDDLQVVLVCVCVVVVADSLQKFWPDWADDV
1ywx_A_1-102      DDKAWDDWDADVPLQKTKTWIKDADD-DDDDAFLVVLVVVCVVVVDDSPQWTFQDKAADP
1xn9_A            DDKDWDDKADDVPLQKIKTWIKDADD-DDADDPVRVLVVVCVVVVHDSLQKDWDDWDADD
2g1d_A_1-98       DAKDKAWAADDDVQGWTKIKIKDDDDDDDDDDLCVCQQNHVVNNVHGSQFWDDDDWDADD

2v94_A_1-93       VHRMIMTIIMGHPDPVSSVVPDDVVRSVSNVD-----------
2v94_B_1-93       VHRMIMTIIMGHPDNVSCVVPPDPVRCVSSPND----------
1ywx_A_1-102      VDRMMTGMTMGHDDVVSCVPVPDCVRCVSVDDDDDDPDDQDDD
1xn9_A            DGRMIITIIMGHPDNVSVCVSVVVVPPDDDDDDDDDDDDDDD-
2g1d_A_1-98       PHHMTIGMTITGPCRVQCVVVNPPGDPVDDDDPPVHPD-----
```

| Statistic | Value |
|---|---|
| Number of structures | 5 |
| Alignment length (columns) | 103 |
| Total positions | 515 |
| Gap fraction | 0.0563 |
| Dropped columns (>50% gaps) | 5 |
| Trimmed alignment length | 98 |
| Average confidence | 0.3512 |
| Average confidence (trimmed) | 0.3651 |

Table S5: Summary statistics for the structure-based alignment shown above.

## Relationship Between Confidence and Gap Fraction

To further understand the drivers of alignment confidence, and as a sanity check on the confidence metric, the relationship between gap fraction (i.e., the proportion of gaps in the full alignment) and the average confidence score was examined. As expected, a strong negative correlation was observed across both datasets and for both alignment methods: alignments with higher gap content consistently exhibit lower

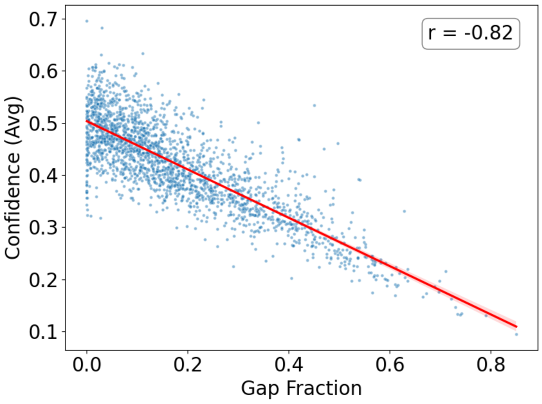


(a) SCOP (ClustalW)

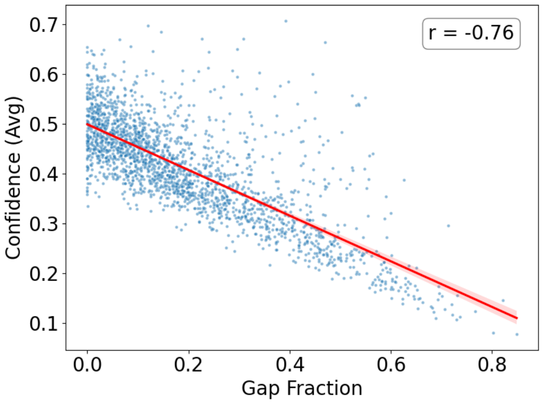


(b) SCOP (MAFFT)

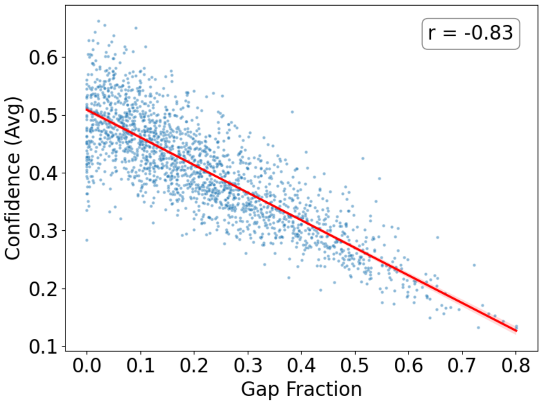


(c) CATH (ClustalW)

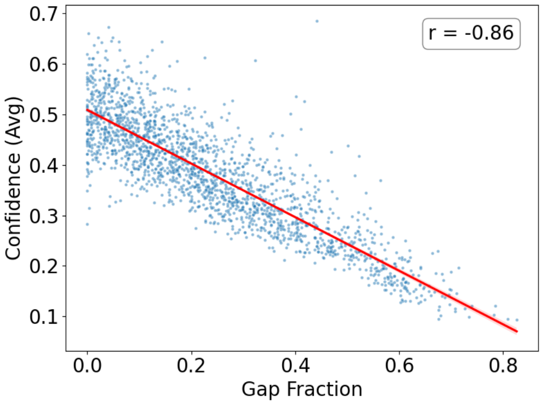


(d) CATH (MAFFT)

Figure S1: Correlation between gap fraction and average alignment confidence. Each point represents a single family alignment from the SCOP and CATH datasets. Panels show the results for alignments generated with both ClustalW and MAFFT. In all cases, a strong negative correlation is observed, confirming that higher gap content is associated with lower overall alignment confidence.

average confidence. Figure S1 shows this inverse relationship for all four combinations of dataset (SCOP, CATH) and alignment method (ClustalW, MAFFT).

These results reinforce the assumption that gaps often reflect regions of structural divergence. A high gap fraction indicates that many sites lack consistently aligned counterparts, whether due to flexible/disordered segments or failure to identify a common structural core. Such columns reduce alignment reliability and introduce

noise in downstream analyses. Thus, removing gap-rich columns, as performed by the tool's trimming function, can effectively reduce poorly supported positions and enrich the alignment for its conserved structural core.

## Utility of the Confidence Score: A Case Study

To provide the full data supporting the case study analysis in the main manuscript, the complete set of phylogenetic trees from both the robustness and negative control sweeps are presented here. The analysis was performed on a dataset of six divergent globin domains with a known ground truth topology, comprising monophyletic pairs of sequences from Bacteria (*C. aurantiacus*, PDB: 5D1V), Archaea (*A. pernix*, PDB: 7UTE), and Eukaryota (*T. newnesi*, PDB: 1T1N).

The following figures are organized into two sections. The first section corresponds to the "robustness test", where phylogenetic trees were inferred from alignments created by progressively removing the lowest-confidence columns. The second section corresponds to the "negative control", where trees were inferred from alignments created by progressively removing the highest-confidence columns. All trees were inferred using IQ-TREE with ultra-fast bootstrap, and the bootstrap support values are shown on the nodes.

## Robustness Test: Progressively Removing Low-Confidence Columns

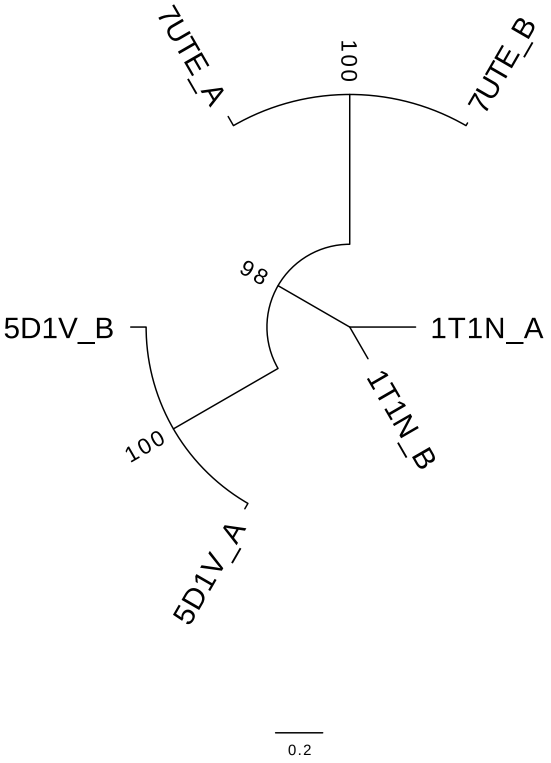


Figure S2: Phylogenetic tree for the globin case study (Robustness Test). This tree was inferred from the alignment after only removing columns with >50% gaps (equivalent to a confidence cutoff > 0.0).

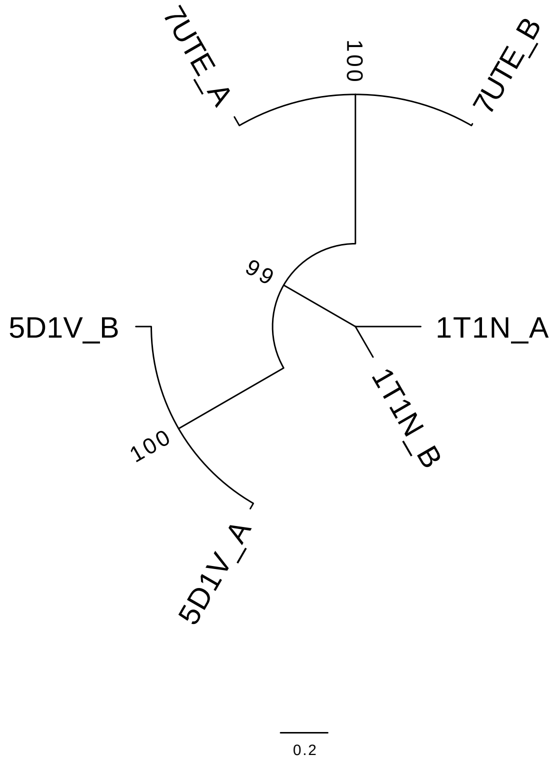


Figure S3: Robustness test continued: Tree inferred from the alignment retaining columns with a confidence score > 0.1.

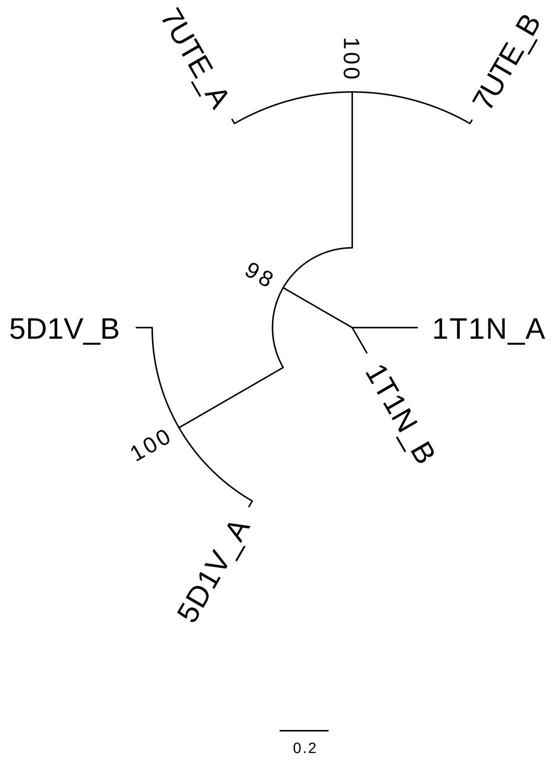


Figure S4: Robustness test continued: Tree inferred from the alignment retaining columns with a confidence score > 0.2.

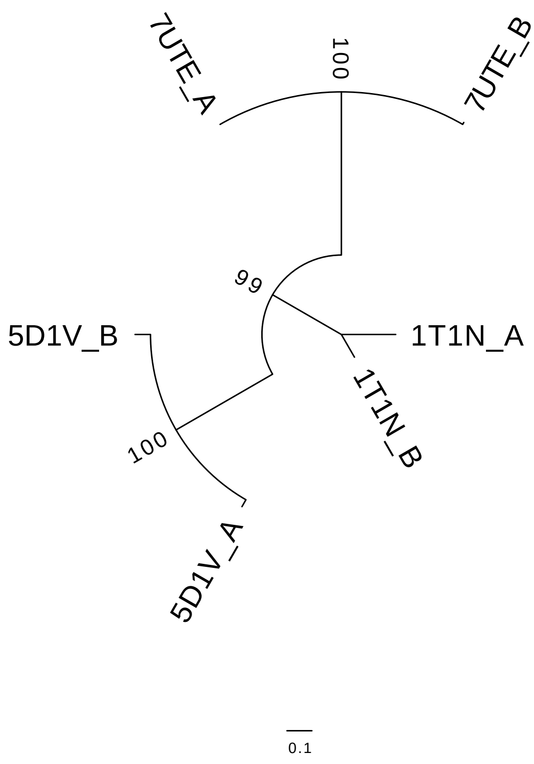


Figure S5: Robustness test continued: Tree inferred from the alignment retaining columns with a confidence score > 0.3.

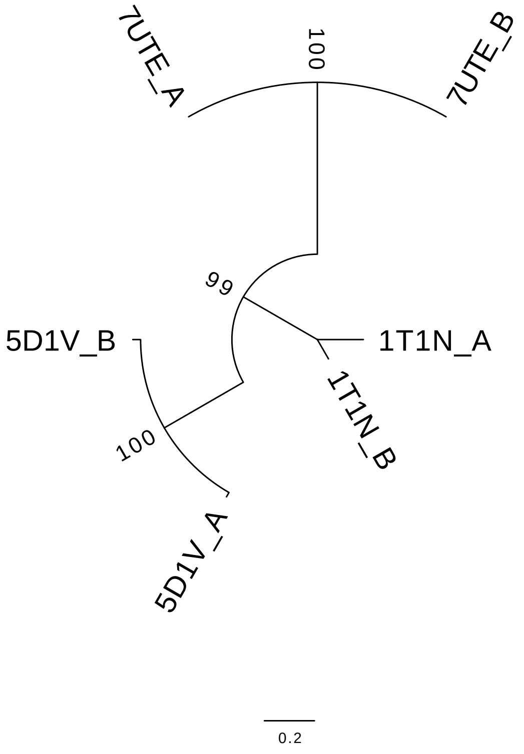


Figure S6: Robustness test continued: Tree inferred from the alignment retaining columns with a confidence score > 0.4.

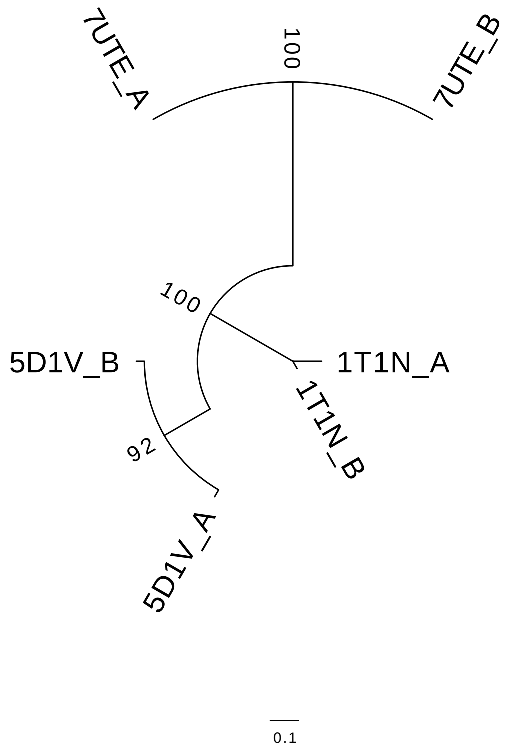


Figure S7: Robustness test continued: Tree inferred from the alignment retaining columns with a confidence score > 0.5.

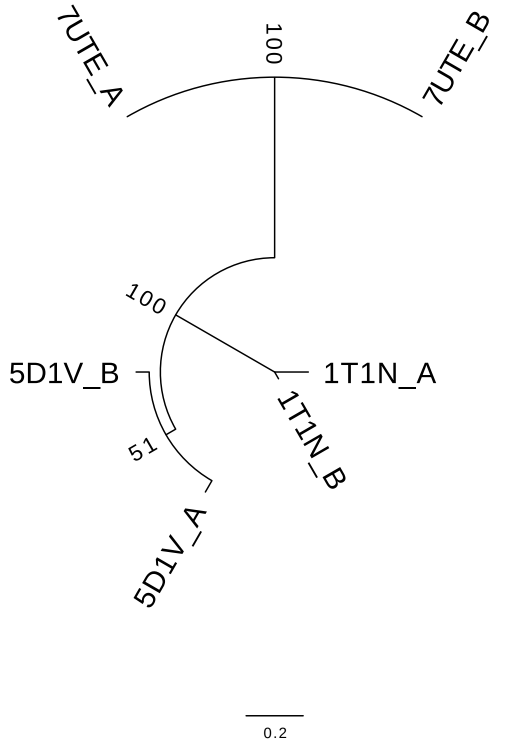


Figure S8: Robustness test continued: Tree inferred from the alignment retaining columns with a confidence score > 0.6.

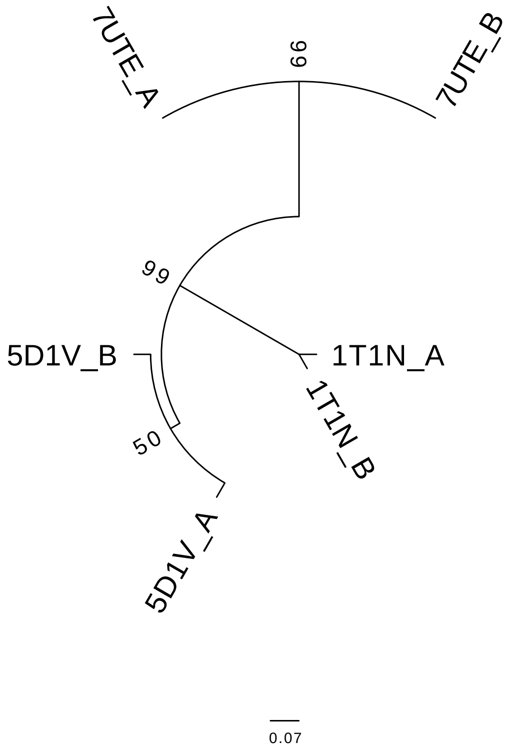


Figure S9: Robustness test continued: Tree inferred from the alignment retaining columns with a confidence score > 0.7.

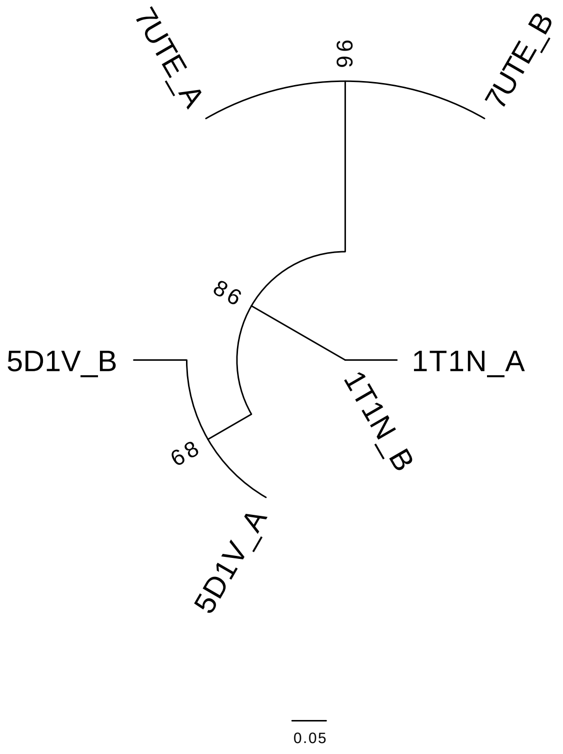


Figure S10: Robustness test continued: Tree inferred from the alignment retaining columns with a confidence score > 0.8.

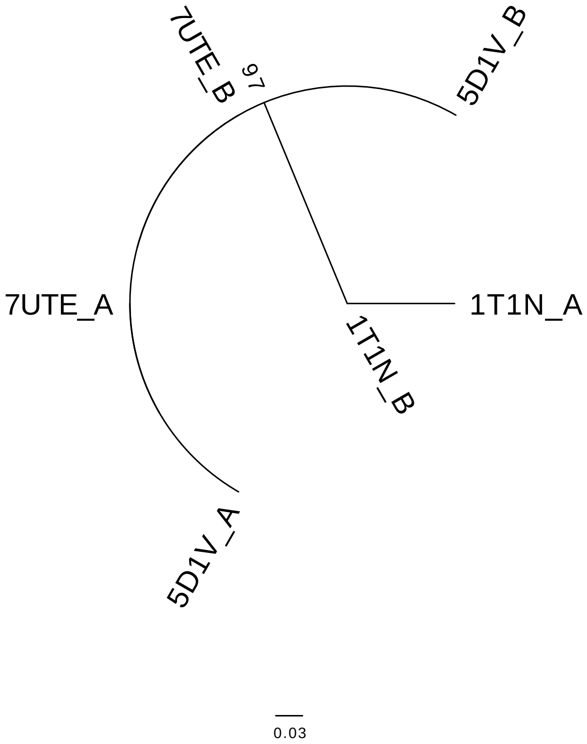


Figure S11: Robustness test continued: Tree inferred from the alignment retaining columns with a confidence score > 0.9. The topology breaks down at this extreme level of trimming.

## Negative Control: Progressively Removing High-Confidence Columns

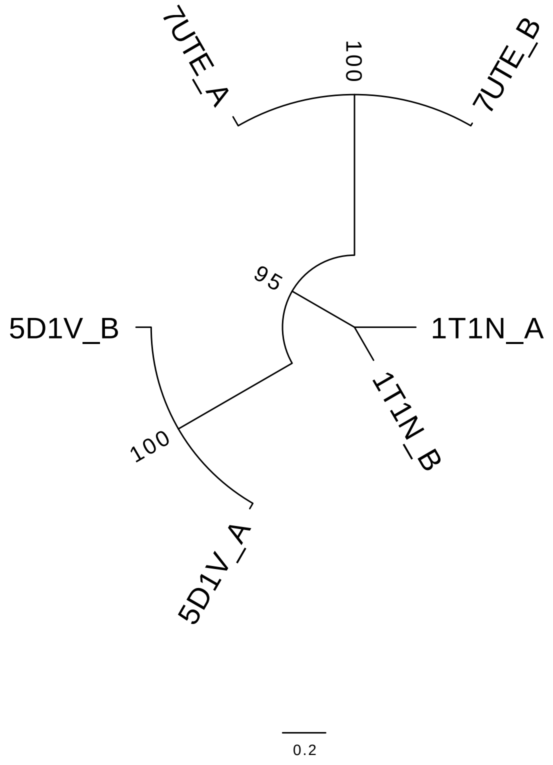


Figure S12: Phylogenetic tree for the globin case study (Negative Control). This tree was inferred from an alignment containing only columns with a confidence score $< 0.9$.

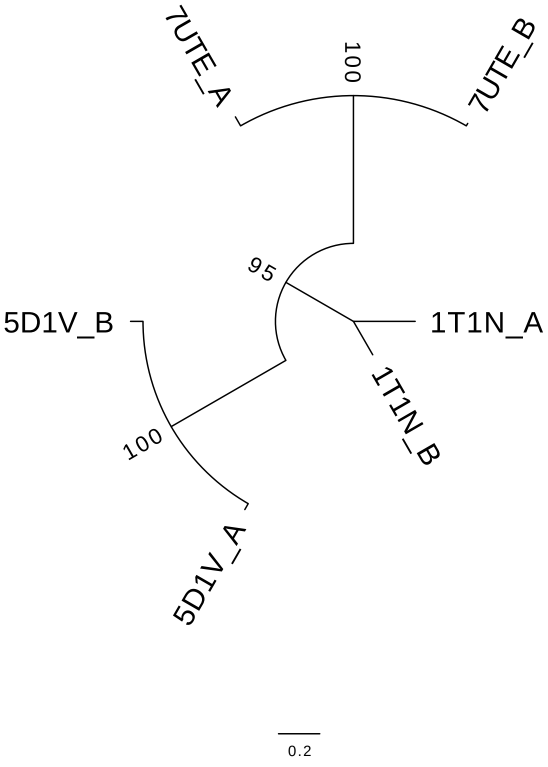


Figure S13: Negative control test continued: Tree inferred from an alignment containing only columns with a confidence score < 0.8.

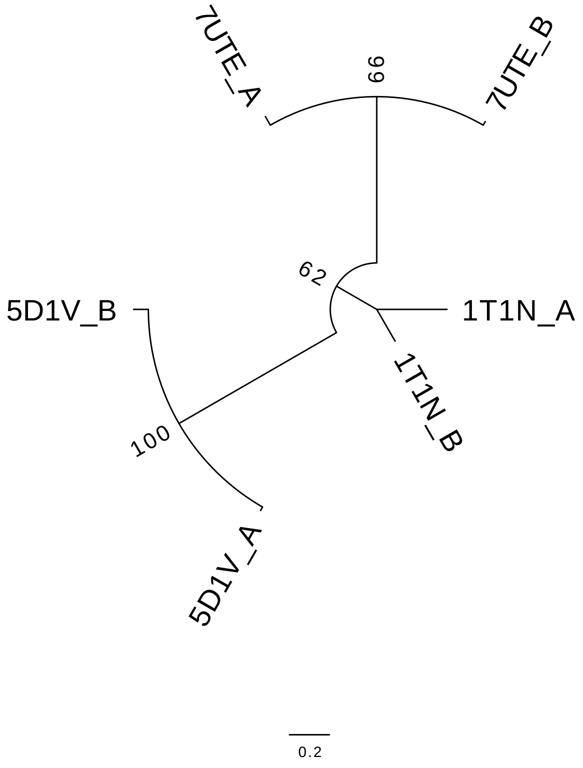


Figure S14: Negative control test continued: Tree inferred from an alignment containing only columns with a confidence score < 0.7.

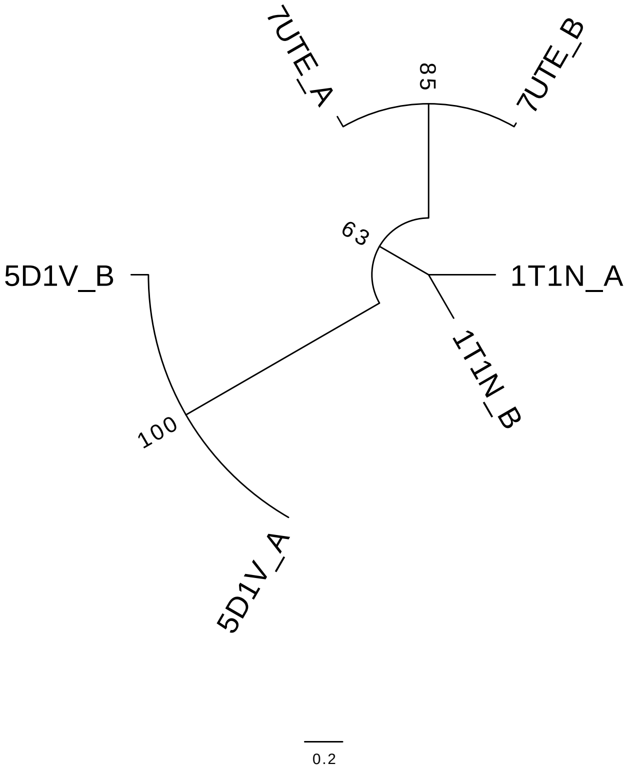


Figure S15: Negative control test continued: Tree inferred from an alignment containing only columns with a confidence score < 0.6.

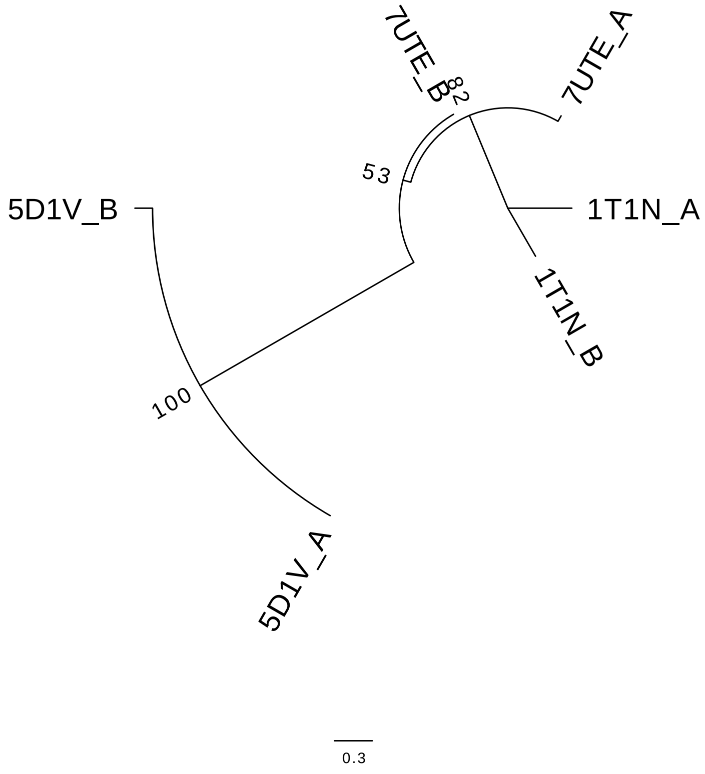


Figure S16: Negative control test continued: Tree inferred from an alignment containing only columns with a confidence score < 0.5.
